# Comparative evaluation of the fecal microbiota of adult hybrid pigs and Tibetan pigs, and dynamic changes in the fecal microbiota of hybrid pigs

**DOI:** 10.3389/fimmu.2023.1329590

**Published:** 2023-12-14

**Authors:** Hengdong He, Yuwei Gou, Bo Zeng, Rui Wang, Jing Yang, Kai Wang, Yunhan Jing, Yuan Yang, Yan Liang, Yuekui Yang, Xuebin Lv, Zhiping He, Qianzi Tang, Yiren Gu

**Affiliations:** ^1^ Key Laboratory of Qinghai-Tibetan Plateau Animal Genetic Resource Reservation and Utilization, Ministry of Education, Southwest Minzu University, Chengdu, Sichuan, China; ^2^ State Key Laboratory of Swine and Poultry Breeding Industry, Key Laboratory of Livestock and Poultry Multi-omics, Ministry of Agriculture and Rural Affairs, and Farm Animal Genetic Resources Exploration and Innovation Key Laboratory of Sichuan Province, College of Animal Science and Technology, Sichuan Agricultural University, Chengdu, Sichuan, China; ^3^ Animal Breeding and Genetics Key Laboratory of Sichuan Province, Sichuan Animal Science Academy, Chengdu, Sichuan, China

**Keywords:** fecal bacteria, Tibetan pigs, bacterial functions, growth stages, host inheritance

## Abstract

The breed of pig can affect the diversity and composition of fecal microbiota, but there is a lack of research on the fecal microbiota of hybrid pigs. In this study, feces samples from Chuanxiang black pigs (a hybrid of Tibetan and Duroc pigs) aged 3 days (n = 24), 70 days (n = 31), 10 months (n = 13) and 2 years (n = 30) and Tibetan pigs aged 10 months (n = 14) and 2 years (n = 15) were collected and sequenced by 16S rRNA gene sequencing technology. We also measured the weight of all the tested pigs and found that the 10-month-old and two-year-old Chuanxiang black pigs weighed about three times the weight of Tibetan pigs of the same age. After comparing the genus-level microbiota composition of Tibetan pigs and Chuanxiang black pigs at 10 months and two years of age, we found that *Treponema* and *Streptococcus* were the two most abundant bacteria in Chuanxiang black pigs, while *Treponema* and *Chirstensenellaceae_R.7_group* were the two most abundant bacteria in Tibetan pigs. Prediction of microbial community function in adult Chuanxiang black pigs and Tibetan pigs showed changes in nutrient absorption, disease resistance, and coarse feeding tolerance. In addition, we also studied the changes in fecal microbiota in Chuanxiang black pigs at 3 days, 70 days, 10 months, and 2 years of age. We found that the ecologically dominant bacteria in fecal microbiota of Chuanxiang black pigs changed across developmental stages. For example, the highest relative abundance of 70-day-old Chuanxiang black pigs at the genus level was *Prevotella*. We identified specific microbiota with high abundance at different ages for Chuanxiang black pigs, and revealed that the potential functions of these specific microbiota were related to the dominant phenotype such as fast growth rate and strong disease resistance. Our findings help to expand the understanding of the fecal microbiota of hybrid pigs and provide a reference for future breeding and management of hybrid pigs.

## Introduction

The development of second-generation sequencing technology has enriched our understanding of human and animal gut microbiota (1). For various agricultural animals, the relationship between digestive tract microbiota and production traits has been attracting increasing interest (2, 3). The large microbiota in the rumen of ruminants, such as cattle and sheep, helps the host digest and absorb forage with high cellulose content (4, 5). The cecum microbiota of single-stomach animals, such as chicken, is found to be associated with weight gain (6, 7). Another study suggests that the domestication process of agricultural animals can affect their gut microbiota in a corresponding way, possibly through shared ecological changes (8). These studies show that the production traits of agricultural animals are closely related to their symbiotic microbiota.

Previous studies revealed that, the production traits of pig, an important agricultural animal, was also impacted by intestinal microbiota (9, 10). Thousands of bacteria have been found to inhabit the pig gut environment, and are widely involved in host growth and development, fat deposition, and other physiological processes (11, 12). The intestinal microbial diversity and composition of pigs changes dynamically with age (13). Piglets begin to be colonized by microbiota after birth and develop intestinal microbiota adapted to breast milk (14). After weaning, the composition of the intestinal microbiota changes dramatically to adapt to this environmental shift (15). However, from weaning to adulthood, the diversity of the intestinal microbiota increases continuously, and intestinal microbiota gradually mature and enter a relatively stable state (16).

In fact, the composition of pig intestinal microbiota depends on a variety of internal and external factors, including breed (host genetics), developmental stage, feeding environment, and feed type (17, 18). An early study found that the pig breed affects the intestinal microbiota composition after controlling for external factors such as environment (19). Significant differences were also found between native Chinese breeds (Meishan, Erhuaface, and Bama) and commercial breeds (Duroc, landrace, and Yorkshire), with the former being more evolutionarily similar (19). These findings were confirmed by subsequent studies (20). The differences between different breeds were reflected in the diversity and the proportion of specific bacteria. For example, the diversity of intestinal microbiota, as measured by the Shannon index, of Jinhua pigs was higher than that of Landrace pigs (21), and *Prevotella* was more abundant in Yorkshire pigs than in Rongchang and Tibetan pigs (22). The latest study conducted 16S rRNA amplification sequencing on the three most common commercial strains (Duroc, Big White, and Yorkshire), and found that although the diversity of the three varieties was similar, the proportion of some bacteria was significantly different (23).

Tibetan pigs are widely bred on the Qinghai–Tibet Plateau in China and are known for their adaptability to low oxygen, cold and drought environment, and coarse feeding (24). Through years of natural selection, Tibetan pigs have formed the following favorable characteristics: (1) high intramuscular fat content, an indicator of meat quality (25), and (2) high-levels of disease resistance and environmental adaptability (26, 27). The harsh environment of the Qinghai–Tibet Plateau has shaped the unique genome and intestinal metagenome of the Tibetan pig (28, 29). To apply the favorable characteristics of Tibetan pigs to efficient commercial production, we crossbred Tibetan and Duroc pigs, and bred the hybrid Chuanxiang black pig, which served as a potential surrogate of the traditional three-way hybrid commercial pig terminal male breed. Such pigs offer the advantages of good quality Tibetan pork, with a high intramuscular fat content and high disease resistance, along with the fast growth rate of Duroc pigs (30). To the best of our knowledge, no study has been conducted on the gut microbes of hybrids between Tibetan pigs and commercial breeds (Big White, Landrace, and Duroc). Considering the unique growth environment of Tibetan pigs, exploring the fecal microbiota composition of Chuanxiang black pigs, a hybrid breed, may provide a new perspective to explore the possible influence of breed on the fecal microbiota. From another aspect, it could also provide reference for breeding and management of hybrid pigs in the future. Therefore, we carried out this study to characterize the differences of fecal microbiota and their potential functions in young adult (10 months) and adult (2 years) between Tibetan pigs and Chuanxiang black pigs. In addition, we also studied the dynamic changes of fecal microbiota for hybrid Chuanxiang black pigs at 3 days of lactation, 70 days of growth, 10 months of young adult and 2 years of adult, and examined specific microbiota and their potential functions across different developmental stages.

## Materials and methods

### Animal management and sample collection

In this study, we used Chuanxiang black pigs, which are a hybrid of Tibetan pigs (6.25% coefficient of relationship) and Duroc pigs (93.75% coefficient of relationship). All Tibetan pigs used for breeding were introduced from Daocheng, Sichuan, China (28°26′N, 99°86′E, 3750m above sea level) in 2003. The Tibetan pigs used in this experiment are their offspring and have adapted to the low-altitude environment. A total of 127 fresh fecal samples were collected from pig farms in Sichuan Academy of Animal Science, Chengdu, Sichuan, China (30°37′N, 104°06′E, 498m above sea level), including Chuanxiang black pigs at 3 days of lactation, 70 days of growth, 10 months of young adult, and 2 years of adult, and Tibetan pigs at 10 months and 2 years age. All fecal samples taken in this experiment were from female Chuanxiang black pigs and female Tibetan pigs. The samples of suckling Chuanxiang black pigs in this experiment were obtained from unrelated litters. The two breeds were raised in the same farm and fed the same feed with a specific nutrient composition (**Supplementary Table 1**). Since Chuanxiang black pigs were bred as boars, with initial mating at 10 months (300 ± 10 days) and middle mating at 2 years (730 ± 10 days), we selected 10 months and 2 years as the young adult and adult sampling points. The young adult and adult Tibetan pigs selected for sampling were of the same age as the Chuanxiang black pigs. The lactation period and post-weaning growth period of pigs are important developmental periods, so 3 days and 70 days were selected as the pre-mature sampling time points for Chuanxiang black pigs. After rectal irritation, individual stool samples were collected in separate sterilized containers, then transported to the laboratory immediately on dry ice and stored at −80°C.

### Weight measurement

All test pigs were weighed in the morning of the same day the fecal samples were taken. The 3-day-old and 70-day-old Chuanxiang black pigs were weighed using a bench scale with model ICS429-BC60 (METTLER TOLEDO, Shanghai, China). All 10-month-old and two-year-old Tibetan pigs and Chuanxiang black pigs were weighed using a cage scale with model WL-B03 (Chengdu Delin Technology Co., Ltd., Chengdu, China).

### DNA extraction and microbial 16S rRNA gene sequencing analysis

The fecal samples were subjected to DNA isolation with an E.Z.N.A.^®^ Soil DNA Kit, following the manufacturer’s instructions. The purity and quality of the genomic DNA were checked using a NanoDrop2000 spectrophotometer (Thermo Fisher Scientific, Waltham, MA, USA). After quantification using the NanoDrop2000 measurements, 30 ng of DNA was used for PCR amplification. The variable region of the 16S rRNA, V4–V5 (515F–907R), was used to design primers as follows: 515F (5′-GTGCCAGCMGCCGCGG-3′) and 907R (5′-CCGTCAATTCMTTTRAGTTT-3′) (31). The PCR products were analyzed using a GeneAmp 9700 PCR system (Applied Biosystems, Foster City, CA, USA). Agarose gel electrophoresis on a 2% gel was used to detect the amplified products of each sample. Purified amplicons were then pooled in equimolar amounts and sequenced on a Biozeron Technology Illumina NovaSeq 2×250 bp paired-end sequencing platform.

16S rRNA gene sequencing data analysis was performed using Quantitative Insights into Microbial Ecology 2 (QIIME2) software (32). The DADA2 method was used for sequence quality control (33). Barcode and primer sequences were removed from the 59 ends, and low-quality bases were truncated from the 39 ends according to the Q20 and Q30 standards. The clean FASTA sequences overlapped, and the feature table was constructed after deredundancy. There were a large number of low abundance features, which would increase the computational workload and affect the FDR correction *P*-value when comparing the differences between high abundance results, and eventually lead to false negative results. According to the total volume of sequencing data, we reserved features with more than five sequence reads. The final high-quality representative feature sequences were used for taxonomic annotation with the SILVA database (138_99 release) (34, 35).

For metrics of taxonomic diversity, we used Shannon indexes to compute the α-diversity. The comparisons of fecal microbiota compositions between the four developmental stages were conducted by univariate permutational analysis of variance (PERMANOVA) for the Bray–Curtis distances and were illustrated by PCoA.

We referred to the SILVA database to obtain the taxonomic composition of microbiota in the QIIME2 analysis process, and default parameters (qiime feature-classifier classify-sklearn –i-reads –i-classifier –o-classification) were used. Linear discriminant analysis (LDA) coupled with linear discriminant analysis effect size (LEfSe) was used to identify differentially abundant taxa among the various developmental stages and between Chuanxiang black pigs and Tibetan pigs (36).

Phylogenetic investigation of communities by reconstruction of unobserved states (PICRUSt2) was used to infer the functional potential of fecal microbiota and we further compared the functional profiles of the two breeds using Statistical Analysis of Metagenomic Profiles (STAMP) software (37, 38).

### Time-series analysis of fecal microbiota in Chuanxiang black pigs

The downstream analysis of time series data included fecal samples from 3 days, 70 days, 10 months, and 2 years for Chuanxiang black pigs. The top 1,000 most abundant features in all samples were selected, and the pipeline described by Coenen et al. was used for follow-up analysis using the R software (39). First, we used the variance Stabilizing Transformation function in the DESeq2 R package to stabilize the variance so that it no longer correlated to species abundance. Then, the Euclidean distance of the species was calculated, and the pam function in the R packet was used for K-Medoids clustering.

We combined clusters with similar trends into a pattern, for example: cluster 1 as pattern 1, clusters 2, 5, and 6 as pattern 2, clusters 3 and 8 as pattern 3, and cluster 4 as pattern 4. Then, the relative abundance of 0.01 was set as a threshold to filter out the low abundance features of each pattern. PICRUSt2 was used to predict the function of high abundance features in each pattern, and the top 20 KEGG enrichment pathways were selected to construct a heatmap using the R package pheatmap.

### Statistical analyses

All experimental data were obtained from at least three independent experiments. The data were statistically analyzed using R (version 4.2.1) software. The Wilcoxon rank-sum test was used to test the significance of the differences between two groups. A significance level of < 0.05 was considered to be statistically significant. na, *P* > 0.05; *, *P*< 0.05; **, *P*< 0.01; ***, *P*< 0.001. To identify genera that had a significant change in different breed, the LDA Effect Size based on the nonparametric Kruskal–Wallis sum-rank test was performed with an alpha value of 0.05 for the factorial Kruskal–Wallis test among classes and a threshold of 4 on the logarithmic LDA score for discriminative features using the galaxy/hutlab website (http://galaxy.biobakery.org/).

## Results

### 16S rRNA sequencing data

In total, we generated 6,764,698 reads from 127 fecal samples. According to the volume of data in the feature table, we only considered features with a total number of sequence reads of more than five. These sequences were subsequently classified as 7,039 bacterial features using the DADA2 program in the QIIME2 platform. We set the sampling depth as 20,000, and found that the number of observed features (**Supplementary Figure 1A**), as well as the Shannon–Wiener index (**Supplementary Figure 1B**), increased with the sampling depth and gradually leveled out, indicating that both the sample size and number of sequence reads were sufficient to cover all bacteria in the community.

The 7,039 features generated by the 16S rRNA sequencing data belonged to the 3-day (lactation), 70-day (growing stage), 10-month-old (young adult), and 2-year-old (adult) Chuanxiang black pigs, and 10-month-old (young adult) and 2-year-old (adult) Tibetan pigs. Next, we analyzed the sequencing data of fecal samples from 10-month-old (young adult) and 2-year-old (adult) Chuanxiang black pigs and Tibetan pigs.

### Differences in diversity between Chuanxiang black pigs and Tibetan pigs at the young adult and adult stages

**Figure 1:**
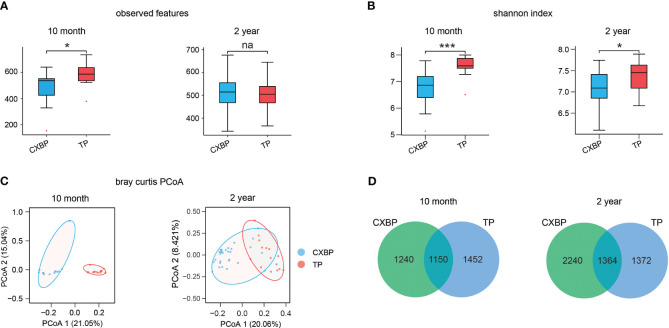
Microbial diversity difference between Chuanxiang black pigs and Tibetan pigs at 10 months and 2 years of age. **(A)** The observed features. **(B)** The Shannon index. **(C)** Principal coordinate analysis (PCoA) based on the weighted Bray–Curtis distances. **(D)** Venn map. In **(A, B)**, small red dots represent outliers. CXBP, Chuanxiang black pig. TP, Tibetan pig. na, *P* > 0.05; *P< 0.05; ***P< 0.001.

According to the boxplot of observable features, 10-month-old Tibetan pigs were significantly different to Chuanxiang black pigs (*P*< 0.05) (**Figure 1A**). By contrast, 2-year-old Tibetan pigs and Chuanxiang black pigs showed a similar number of observable features (*P* = 0.58) (**Figure 1A**). In terms of α-diversity represented by the Shannon index, Tibetan pigs showed higher α-diversity than Chuanxiang black pigs at both 10 months (*P*< 0.001) (**Figure 1B**) and 2 years (*P*< 0.05) of age (**Figure 1B**). The principal coordinate analysis (PCoA) map based on the Bray–Curtis distance showed that the community members and structure of Chuanxiang black pigs and Tibetan pigs at 10 months and 2 years ranged from completely different to partially similar (**Figure 1C**). Among all of the features observed at 10 months, 1,150 features were shared by the two types of pigs, and another 1,240 and 1,452 features were unique to Chuanxiang black pigs and Tibetan pigs, respectively. Among all of the features observed at 2 years, 1,364 were common to the two species of pigs, and another 2,240 and 1,372 were unique to Chuanxiang black pigs and Tibetan pigs, respectively (**Figure 1D**).

### Differences in bacteria in the fecal microbiota between Chuanxiang black pigs and Tibetan pigs

**Figure 2:**
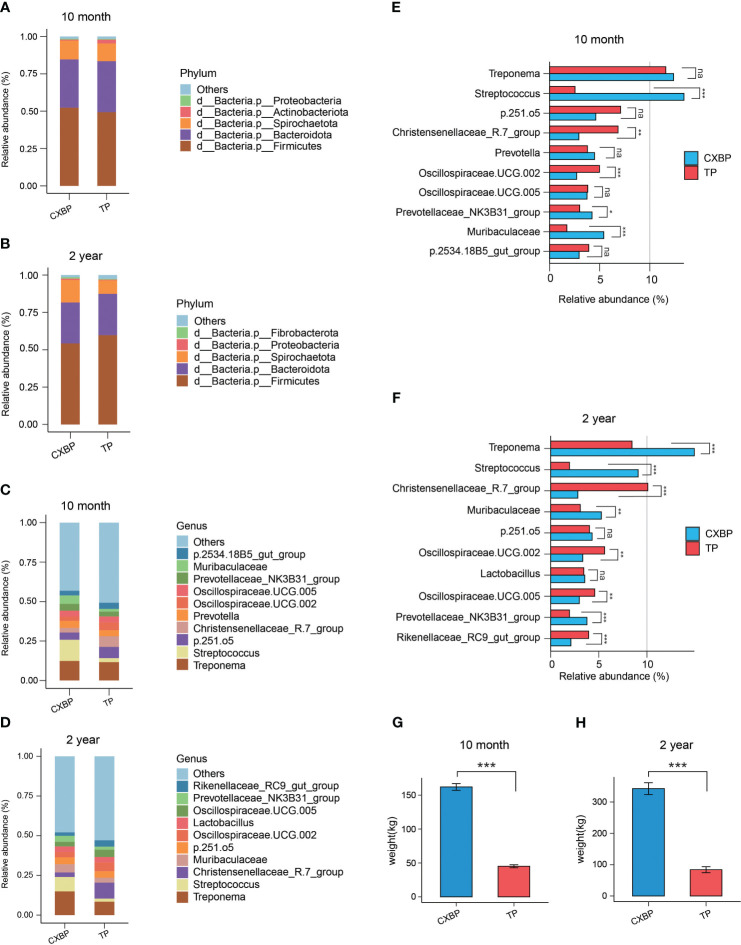
The microbial composition at the phylum and genus levels, and the weight data. **(A)** At the phylum level, the top 5 most abundant microbes in fecal samples of Chuanxiang black pigs and Tibetan pigs at 10 months of age; phyla with a lower relative abundance are classified as “Others”. **(B)** At the phylum level, the top 5 most abundant microbes in fecal samples of Chuanxiang black pigs and Tibetan pigs at 2 years of age. Phyla with a lower relative abundance were classified as “Others”. **(C)** At the genus level, the top 10 most abundant microbes in fecal samples of Chuanxiang black pigs and Tibetan pigs at 10 months of age. Genera with a lower relative abundance are classified as “Others”. **(D)** At the genus level, the top 10 most abundant microbes in fecal samples of Chuanxiang black pigs and Tibetan pigs at 2 years of age; genera with a lower relative abundance are classified as “Others”. **(E)** The top 10 most abundant microbes at genus level in fecal samples of Chuanxiang black pigs and Tibetan pigs at 10 months of age were shown as bar chart. **(F)** The top 10 most abundant microbes at genus level in fecal samples of Chuanxiang black pigs and Tibetan pigs at 2 years of age were shown as bar chart. **(G)** Average weight data of Chuanxiang black pigs and Tibetan pigs at 10 months of age. The error bar represents the standard deviation. **(H)** Average weight data of Chuanxiang black pigs and Tibetan pigs at 2 years of age. The error bar represents the standard deviation. CXBP, Chuanxiang black pig. TP, Tibetan pig. na, *P* > 0.05; *P< 0.05; **P< 0.01; ***P< 0.001.

We classified features to the phylum and genus levels, and detected the composition of the fecal microbiota for Chuanxiang black pigs and Tibetan pigs of distinct ages at the different taxonomic levels. At the phylum level, a total of 22 phyla of fecal microbiota were found in 10-month-old Chuanxiang black pigs and Tibetan pigs (**Supplementary Table 2**), and a total of 24 phyla of fecal microbiota were found in 2-year-old Chuanxiang black pigs and Tibetan pigs (**Supplementary Table 3**). Firmicutes was the most abundant phylum followed by Bacteroidetes and Spirochaetota across the two adult stages and the two breeds (**Figures 2A, B**). We found that the fecal microbiota composition of Chuanxiang black pigs and Tibetan pigs was highly similar at the phylum level (**Figures 2A, B**).

At the genus level, we showed the relative proportions of the top 10 bacterial genera in the fecal microbiota of Chuanxiang black pigs and Tibetan pigs across the two adult stages (**Figures 2C, D**; **Supplementary Figure 2**). *Treponema* and *Streptococcus* ranked as the top 2 genera in relative abundance for Chuanxiang black pigs at the age of 10 months and 2 years (**Figures 2C, D**), and *Treponema* and *Chirstensenellaceae_R.7_group* ranked as the top 2 genera in relative abundance for Tibetan pigs at the age of 10 months and 2 years (**Supplementary Figure 3**). Among the top 10 bacterial genera in the pigs at the age of 10 months, the proportion of *Treponema* was different between the two pig breeds (*P*= 0.35) (**Figure 2E**), the proportion of *Streptococcus* in the fecal microbiota of Chuanxiang black pigs was significantly higher than that of Tibetan pigs (*P*< 0.001) (**Figure 2E**), and the proportion of *Chirstensenellaceae_R.7_group* in the fecal microbiota of Tibetan pigs was significantly higher than that of Chuanxiang black pigs (*P* < 0.01) (**Figure 2E**). These trends changed for pigs at the age of 2 years (**Figure 2F**): the proportion of *Treponema* in the fecal microbiota of Chuanxiang black pigs was higher than that of Tibetan pigs (*P*< 0.001) (**Figure 2F**), the proportion of *Streptococcus* in the fecal microbiota of Chuanxiang black pigs remained higher than that of Tibetan pigs (*P*< 0.001) (**Figure 2F**), and the proportion of *Chirstensenellaceae_R.7_group* in the fecal microbiota of Tibetan pigs was significantly higher than that of Chuanxiang black pigs (*P*< 0.001) (**Figure 2F**). It is worth noting that the body weight of 10-month-old Chuanxiang black pigs is more than three times that of Tibetan pigs (*P*< 0.001), and the 2-year-old pigs show a similar phenomenon (*P*< 0.001) (**Figures 2G, H**).

**Figure 3:**
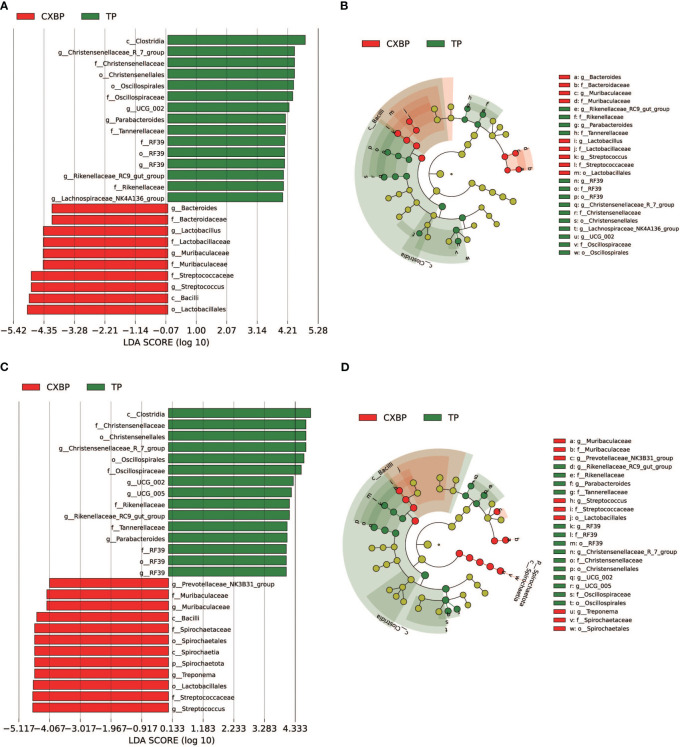
Comparison of the fecal microbiota between Chuanxiang black pigs and Tibetan pigs at 10 months and 2 years of age. **(A, B)** are LEfSe analysis charts at 10 months, LDA > 4. **(C, D)** are LEfSe analysis charts at 2 years, LDA > 4. In the cladistic diagram of Figures **(B, D)**, the circles radiating from the inside out represent the taxonomic rank of different microbiota from phylum to genus. Each small circle in the different taxonomic rank represents a taxonomic rank below that level, and the diameter of the small circle represents its relative abundance. CXBP, Chuanxiang black pig. TP, Tibetan pig.

To determine the specific bacteria in the fecal microbiota of Chuanxiang black pigs and Tibetan pigs, linear discriminant analysis effect size (LEfSe) was employed to analyze the fecal microbiota, and linear discriminant analysis (LDA) was used to further determine the differences in fecal microbiota composition between Chuanxiang black pigs and Tibetan pigs at the ages of 10 months and 2 years. Analyses of pigs at the age of 10 months showed that the concentrations of *Clostridia* and *Chirstensenellaceae_R_7_group* were higher in the fecal microbiota of Tibetan pigs, whereas those of *Lactobacillales* and *Bacilli* were increased for Chuanxiang black pigs (LDA > 4) (**Figures 3A, B**). At the age of 2 years, the concentrations of *Clostridia* and *Chirstensenellaceae* were higher in Tibetan pigs, while those of *Lactobacillales* and *Streptococcus* were elevated in Chuanxiang black pigs (LDA > 4) (**Figures 3C, D**).

### Prediction of function of the fecal microbiota of Tibetan pigs and Chuanxiang black pigs at the two adult stages

**Figure 4:**
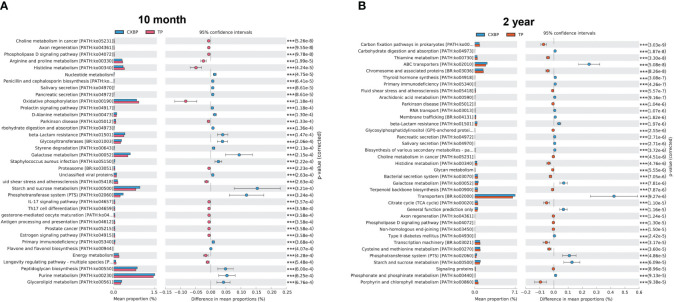
Prediction of functional pathways for fecal microbiota. **(A)** The significantly differential pathways between Chuanxiang black pigs and Tibetan pigs at 10 months of age (*P*< 0.001). **(B)** The significantly differential pathways between Chuanxiang black pigs and Tibetan pigs at 2 years of age (*P* < 0.0001). CXBP, Chuanxiang black pig. TP, Tibetan pig. ***P< 0.001.

We used PICRUSt2 to predict the function of the fecal microbiota of Chuanxiang black pigs and Tibetan pigs at the ages of 10 months and 2 years, and obtained the differential KEGG enrichment pathways for the two breeds at these two adult stages. For 10-month-old pigs, beta-lactam resistance, glycosyltransferases, and galactose metabolism were enriched in Chuanxiang black pigs, while the over-represented pathways for Tibetan pigs included arginine and proline metabolism, histidine metabolism, and oxidative phosphorylation (*P*< 0.001) (**Figure 4A**). For 2-year-old pigs, ABC transporters, beta-lactam resistance, galactose metabolism, and other enrichment pathways were enriched in Chuanxiang black pigs, while the over-represented pathways for Tibetan pigs included carbon fixation pathways in prokaryotes, thiamine metabolism, and chromosome-associated proteins (*P*< 0.0001) (**Figure 4B**).

### Dynamic changes in fecal microbiota across the postnatal developmental stages in Chuanxiang black pigs

**Figure 5:**
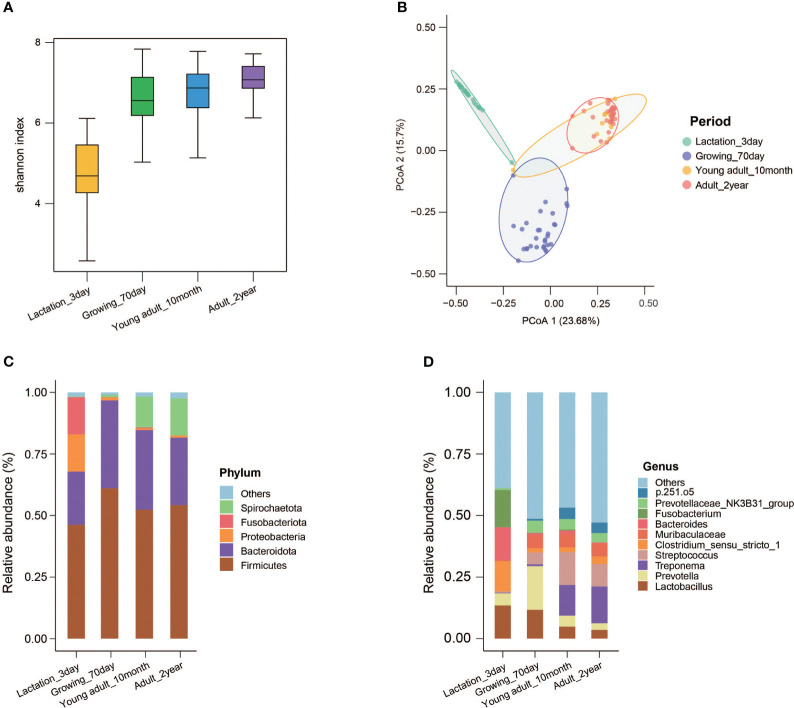
Microbial composition and diversity of Chuanxiang black pigs at different developmental stages. **(A)** The Shannon index. **(B)** Principal coordinate analysis (PCoA) based on the weighted Bray–Curtis distances. **(C)** The top five microbes in relative abundance in feces samples at the phylum level; the phyla with a lower relative abundance are classified as “Others”. **(D)** The top 10 microbes in relative abundance in feces samples at the genus level; the genera with a lower relative abundance are classified as “Others”.

We measured the weight of each pig at different stages of development (**Supplementary Table 4**). The α-diversity (represented by the Shannon index) of Chuanxiang black pigs gradually increased over the developmental process, and the change was particularly evident from 3 days to 70 days (**Figure 5A**). PCoA based on Bray–Curtis distances showed that the fecal microbiota structure at 3 days and 70 days were distinct, while the fecal microbiota structure at 10 months and 2 years clustered in close proximity (**Figure 5B**). At the phylum level, we observed that Firmicutes and Bacteroidetes were the most abundant across distinct developmental stages. Proteobacteria and Fusobacteriota accounted for approximately 30% of the total sequences for pigs at the age of 3 days (**Supplementary Table 5**). Spirochaetota became the third most dominant phylum at the adult stages (10 months and 2 years) (**Figure 5C**). At the genus level, the top 10 bacterial genera in relative abundance were *Lactobacillus*, *Prevotella*, *Treponema*, *Streptococcus*, *Clostridium_sensu_stricto_1*, *Muribaculaceae*, *Bacteroides*, *Fusobacterium*, *Prevotellaceae_NK3B31_group* and *p.251.o5*. The percentage of *Lactobacillus* decreased gradually over the developmental stages. *Prevotella* became the first dominant bacterium in pigs at the age of 70 days, while the contribution of *Prevotella* was negligible at other developmental stages. *Treponema* and *Streptococcus* showed low abundance in the lactation and growth stages, but ranked first and second in the adult stages. *Sensu_sensu_stricto_1*, *Bacteroides*, and *Fusobacterium* were the dominant bacteria in 3-day-old piglets (**Figure 5D**; **Supplementary Figure 4**).

### Fecal microbiota specific to distinct developmental stages for Chuanxiang black pigs and their functions

**Figure 6:**
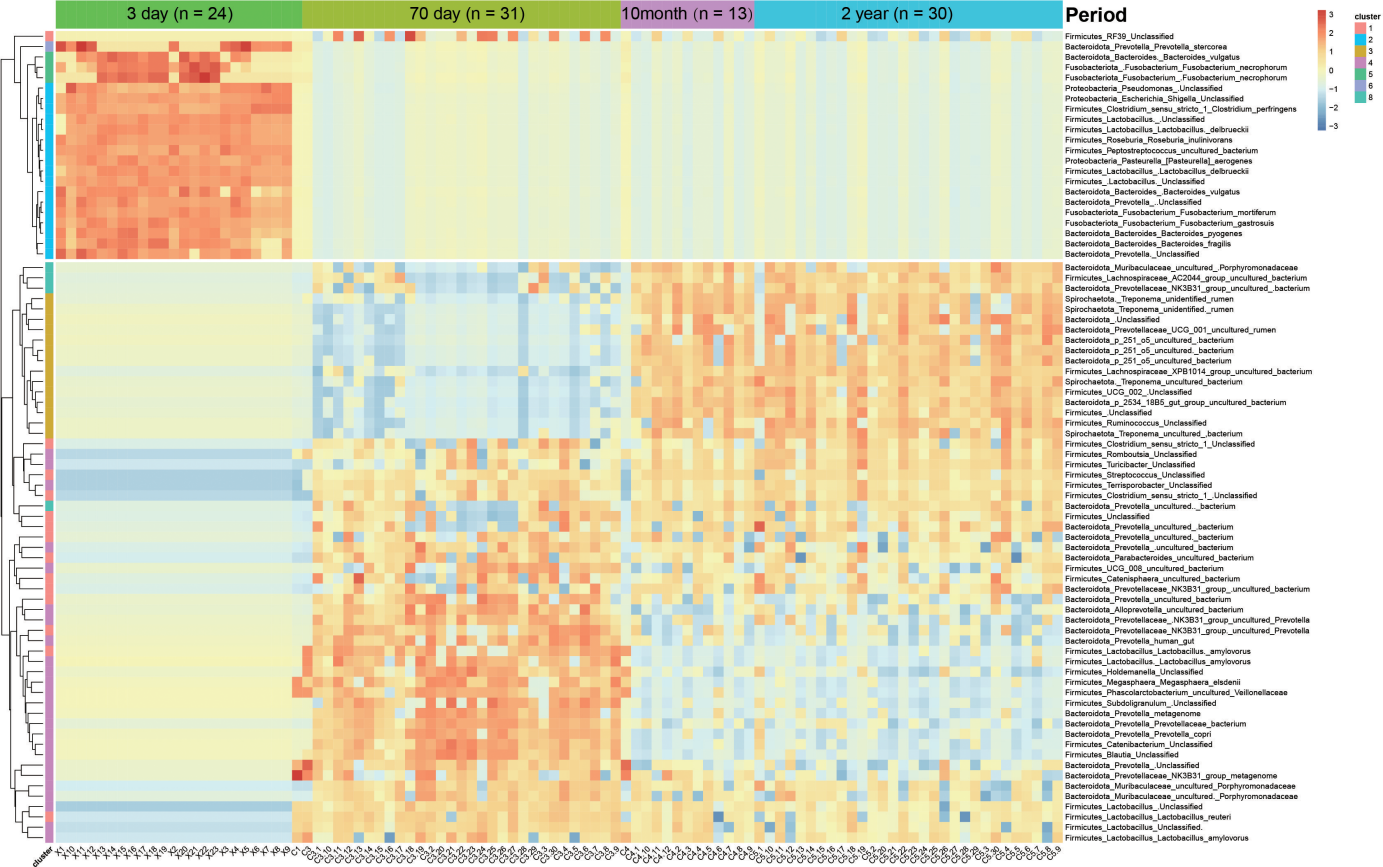
Heatmap of developmental stage-specific features. Rows indicate the “phylum level_genus level _species level” classification results.

We selected the top 1,000 most abundant features and obtained eight clusters by K-Medoids clustering based on the characteristics of phase changes. Among them, the features of clusters 2, 5, and 6 were significantly higher for Chuanxiang black pigs at the lactation stage compared with the other stages. Since the abundance of these three clusters was low for the other stages, we defined these three clusters as lactation-specific microbiota. Similarly, clusters 3 and 8 were defined as 10-month- and 2-year-specific microbiota, and cluster 4 was defined as long-term 70-day-specific microbiota. Cluster 1 was defined as an fecal “frequent visitor” because of its consistently high abundance across the ages of 70 days, 10 months, and 2 years. Cluster 7 did not exhibit any stage specificity (**Supplementary Table 6**). We summarized the features with an average relative abundance of greater than 1% for each cluster, classified them at the species level, and displayed the findings using heatmaps (**Figure 6**). We noted that four features of *Fusobacterium* and four features of *Bacteroides* were classified into clusters 2, 5, and 6. A variety of *Prevotella* features were classified into cluster 4. Clusters 3 and 8 contained the high abundance features of four species of *Treponema*. However, only one feature in the genus *Streptococcus* was retained after filtering for low abundance and was classified as cluster 1. We then predicted the PICRUSt2 function of these selected high-abundance features specific to distinct stages, and showed the enriched KEGG pathways with the top 20 significance (**Supplementary Figures 5A–D**). We found that the over-represented functional categories of specific microbiota changed from nucleic acid metabolism and other pathways related to cell processes at lactation, to nutrient absorption at the growth stage, and DNA repair and other pathways related to cell renewal were involved at the young adult and adult stages (**Supplementary Figures 5A–D**).

## Discussion

We found that Firmicutes, Bacteroidetes and Spirochaetota have always been the dominant phylum in fecal microbiota of 10-month-old and 2-year-old Chuanxiang black pigs and Tibetan pigs. Firmicutes and Bacteroidetes are consistently reported to be the two predominant phyla in most reports on pig intestinal microbiota (40). Spirochaetota is also present in most reports on the microbiota of Tibetan pigs (41). At the genus level, *Treponema* was present in high abundance in both Tibetan pigs and Chuanxiang black pigs. A previous study reported high rates of *Treponema* in the gut of the hunter-gatherer Hadza people, which improves field fitness and adaptation to a high-fiber diet (42). Our results imply that Tibetan pigs, as a wild grazing breed, also have higher *Treponema* in their feces. Since Tibetan pigs and Chuanxiang black pigs were uniformly fed commercial diets with a low cellulose content in the experiment, the influence of diet on *Treponema* could be excluded. We hypothesize that Chuanxiang black pigs may have inherited from their Tibetan ancestors bacteria that improve resistance to rough feeding and host environmental adaptability. The abundance of *Streptococcus* was higher in Chuanxiang black pigs than in Tibetan pigs, both at the ages of 10 months and 2 years. *Streptococcus* is generally considered to be a pathogenic bacterium associated with various inflammatory reactions (43). As reported by a previous study, *Streptococcus* was 2–3 times more prevalent in the intestinal tract of Duroc pigs than in Large White and Landrace pigs on days 123 and 158 (23), and similarly, the content of *Streptococcus* was lower in Tibetan pigs compared with Duroc pigs (41). One study also found that the content of *Streptococcus* was higher in pigs with a faster growth rate and proved to be a strong probiotic candidate (13). We speculated that *Streptococcus* in Chuanxiang black pigs is derived from its Duroc ancestors. The phenomenon occurs after adulthood, rather than during the relatively fragile early stages of development (44). Therefore, we speculate that a high content of *Streptococcus* could contribute to the growth of Chuanxiang black pigs, while at the same time, may maintain the intestinal tract in a state of “vigilance”, helping in the prevention of inflammation (45). In addition, we also noted that *Chirstensenellaceae_R.7_group* was significantly more abundant in Tibetan pigs at the age of 10 months than in Chuanxiang black pigs, and this trend was more evident at the age of 2 years. In humans, low-energy diets lead to a significant increase in the relative abundance of *Chirstensenellaceae_R.7_group* (46). *Chirstensenellaceae_R.7_group* belongs to the family *Chirstensenellaceae*, members of which are associated with a high-fiber and high-protein diet and are considered to be the signature flora of host health (47). In addition, its high heritability has also been demonstrated (48). The relative proportion of *Chirstensenellaceae_R.7_group* jumped to first place when Tibetan pigs reached 2 days of age, which has not been reported previously. We propose that the high proportion of *Chirstensenellaceae_R.7_group* is a sign of the healthy and mature fecal microecology of Tibetan pigs. The relative proportion of *Chirstensenellaceae_R.7_group* in Chuanxiang black pigs during the 2-year period was obviously lower than that of Tibetan pigs, although it ranked in the top 10. These results indicated that the Tibetan pig pedigree affected the *Chirstensenellaceae_R.7_group* population in the feces of Chuanxiang black pigs. However, because of the obvious negative correlation between *Chirstensenellaceae_R.7_group* and body mass index (47), the abundance of *Chirstensenellaceae_R.7_group* may not be as high in the feces of Chuanxiang black pigs as Tibetan pigs. This phenomenon is consistent with our results on body weight, namely, that the body weight of Chuanxiang black pigs at 10 months and 2 years of age is three times that of Tibetan pigs.

Previous studies reported that the intestinal microbiota of pigs with faster weight gain is also more conducive to carbohydrate and lipid absorption (49), which is consistent with the enriched functional categories of microbiota over-represented in adult Chuanxiang black pigs compared to Tibetan counterparts. In contrast, young adult and adult Tibetan pigs showed higher enrichment in functions such as immune response regulation and roughage tolerance, which was consistent with the results of Niu et al. (41). We hypothesized that the significant difference in functional prediction of fecal microbiota between Chuanxiang black pigs and Tibetan pigs at young adult and adult stages may be related to the microbiota of their Duroc ancestors.

The above results indicate the successful construction of the hybrid Chuanxiang black pig from the perspective of fecal microbiota. We were particularly interested in the characteristics of the microbiota at different developmental stages in Chuanxiang black pigs and whether the microbiota was associated with advantageous properties. We found that as the developmental stage progressed, the α-diversity of the fecal microbiota of Chuanxiang black pigs gradually increased, which was consistent with the findings of previous studies (13). The age of commercial pigs is generally about 180 days, and our results confirmed that the fecal microbiota of pigs maintained a relatively stable state after 5–6 months (50). After classifying the features to the phylum level, we observed that Proteobacteria and Fusobacteria accounted for approximately 30% of the fecal microbiota for piglets at 3 days age, and these two phyla were almost completely absent at later stages of development, consistent with a previous study on Tibetan pigs (41). Proteobacteria is a well-recognized phylum associated with microecological imbalance and disease risk (51). The increased abundance of Proteobacteria, including in patients with cirrhosis and hepatitis, has revealed a high correlation with disease occurrence (52). In another review, Kelly et al. reported that Fusobacteria activates host inflammatory responses, designed to protect the host from pathogens that promote tumor growth (53). Furthermore, a recent study found that the abundance of Fusobacteria in the intestinal tract of Tibetan pigs with diarrhea was significantly higher than that in healthy Tibetan pigs and in piglets after diarrhea (54). Fusobacteria and Proteobacteria appear in a high abundance in newborn piglets. Whether the specific role of Fusobacteria and Proteobacteria is beneficial or harmful remains to be further explored. However, what concerned us was the change at the genus level. First, *Lactobacillus*, a probiotic that is known to aid host growth and fight infection, dominates during lactation, and then abundance gradually decreases (55). Second, *Fusobacterium*, belonging to the phylum Fusobacteria, has been reported to be significantly increased in abundance in piglets with epidemic diarrhea, which is consistent with the properties of Fusobacteria (56). The high abundance of *Fusobacterium* suggests that the diet and environment of piglets should be carefully managed to prevent acute diarrhea. In addition, we found that several bacteria specific to newborn piglets, including *Bacteroides* and *Clostridium sensustricto 1*, exist in high abundance, which was consistent with previous studies (57). However, different from previous studies, the relative abundance of *Prevotella*, which is low in the intestinal tract of Tibetan pigs, increased significantly during the growth stage in Chuanxiang black pigs, which was more likely to occur in commercial pig breeds such as Duroc according to previous reports (13, 41). In a study of 698 Duroc pigs and their intestinal flora, Chen et al. indicated that *Prevotella* is associated with excessive energy intake and is considered to be the core genus of bacteria formed after prolonged feeding with a high-energy and high-protein diet in commercial pigs (58). The high abundance of *Prevotella* may be closely related to the high growth rate of Chuanxiang black pigs compared with Tibetan pigs. The characteristics and potential functions of *Treponema* and *Streptococcus*, as discussed above, indicate the possibility that *Treponema* and *Streptococcus* were inherited from Tibetan pigs and Duroc pigs, respectively. During the developmental process of Chuanxiang black pigs, the fecal microbiota composition of Chuanxiang black pigs was found to be similar to that of Tibetan and Duroc counterparts, for piglets at lactation and growth stages, respectively (23, 41). Interestingly, the fecal microbiota composition of Chuanxiang black pigs at the adult stage showed both characteristics of Tibetan pigs and Duroc counterparts (23). The improved disease resistance and meat quality of newborn and adult Chuanxiang black pigs and the higher growth rate of young and adult Chuanxiang black pigs also corresponding to these findings (30). Therefore, these phenomena reveal that the superior characteristics of Chuanxiang black pigs is related to the evolution and composition of the fecal microbial community. Conversely, the ancestral or parental Duroc and Tibetan pigs of Chuanxiang black pigs might also affect the changes in the fecal microbial community.

Although technical limitations indicate that it is unreliable to classify 16S rRNA amplicons at the species level (59), some of the results of the specific microbiota we identified at the feature level agree with previous studies (13), and also confirms many of our findings at the genus level. For example, “*Prevotella copri*” with high abundance was a 70-days-specific microbe and belongs to the genus *Prevotella* of the phylum Bacteroidota, which was consistent with the results obtained by Chen et al. in Duroc at the same developmental stage (58). Our functional prediction results for the stage-specific microbiota of Chuanxiang black pigs is also consistent with their potential function, reflecting each unique stage of the host to some extent (13). In addition, the specific microbiota may affect the physiological function of Chuanxiang black pigs through the release of certain metabolites (60).

The limitation of this study is that we were unable to collect and include feces samples from Duroc at different developmental stages and at 3 and 70 days of Tibetan pigs under the same feeding conditions. In addition, this study did not explore host-microbial interactions at the genetic level. In the future, better experimental design and multi-level sequencing data are needed to explain the changes in fecal microbiota in hybrid pigs at different developmental stages.

In conclusion, this study revealed the differences in fecal microbiota between adult Chuanxiang black pigs and Tibetan pigs. There were obvious changes in the microbial composition of the two pig breeds, which may be related to the Chuanxiang black pig being a crossbreed between Duroc and Tibetan pigs. In addition, the fecal microbial diversity and composition of Chuanxiang black pigs showed dynamic changes during developmental growth. We speculate that the emergence of some specific strains of fecal microbiota at particular developmental stages may have influenced the formation of favorable characteristics. These results contribute to better understanding of fecal microbiota in hybrid pigs and provide reference for breeding and management of hybrid pigs in the future.

## Data availability statement

The datasets presented in this study can be found in online repositories. The names of the repository/repositories and accession number(s) can be found below: https://www.ncbi.nlm.nih.gov/, PRJNA981435.

## Ethics statement

The complete procedure in this study was approved by the Committee on the Care and Use of Laboratory Animals of the State-Level Animal Experimental Teaching Demonstration Center of Sichuan Agricultural University.

## Author contributions

HDH: Formal Analysis, Investigation, Methodology, Software, Visualization, Writing – original draft. YWG: Methodology, Software, Visualization, Writing – original draft. BZ: Formal Analysis, Investigation, Supervision, Writing – review & editing. RW: Formal Analysis, Methodology, Writing – original draft. JY: Formal Analysis, Investigation, Writing – original draft. KW: Investigation, Writing – original draft. YHJ: Investigation, Writing – original draft. YY: Investigation, Writing – original draft. YL: Data curation, Formal Analysis, Writing – original draft. YKY: Data curation, Investigation, Writing – original draft. XBL: Data curation, Investigation, Writing – original draft. ZPH: Conceptualization, Funding acquisition, Project administration, Resources, Supervision, Writing – review & editing. QZT: Conceptualization, Funding acquisition, Methodology, Project administration, Resources, Software, Supervision, Validation, Writing – review & editing. YRG: Conceptualization, Data curation, Funding acquisition, Investigation, Project administration, Resources, Supervision, Validation, Writing – review & editing.
