# Supplementary material for: Comparative evaluation of the fecal microbiota of adult hybrid pigs and Tibetan pigs, and dynamic changes in the fecal microbiota of hybrid pigs
Source: Front Immunol. 2023 Dec 14;14:1329590. doi: 10.3389/fimmu.2023.1329590 (PMC10752980; doi:10.3389/fimmu.2023.1329590)
Supplement: Supplementary file 2 [file Image_1.pdf]

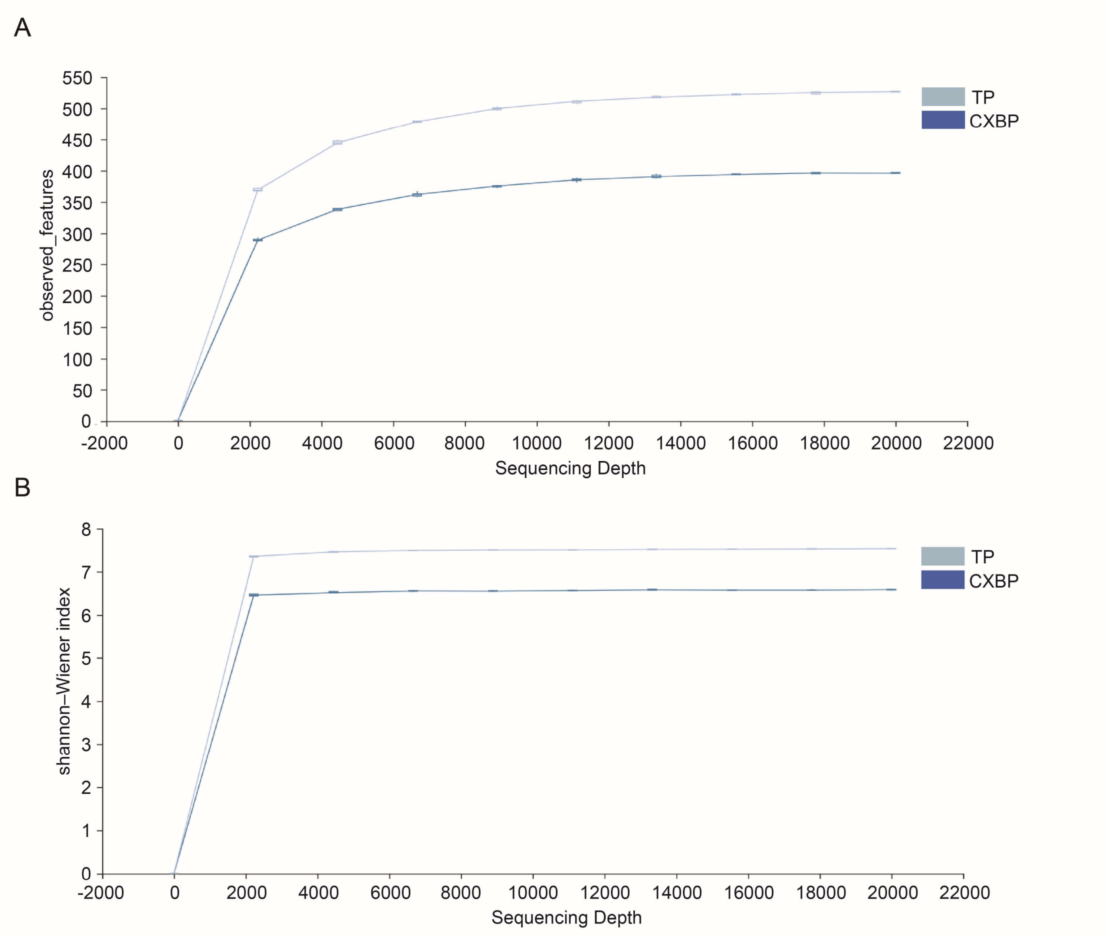

Supplementary Figure 1. The number of observed features (A) and the Shannon–Wiener index (B) with deepening sampling depth. CXBP, Chuanxiang black pig. TP, Tibetan pig.

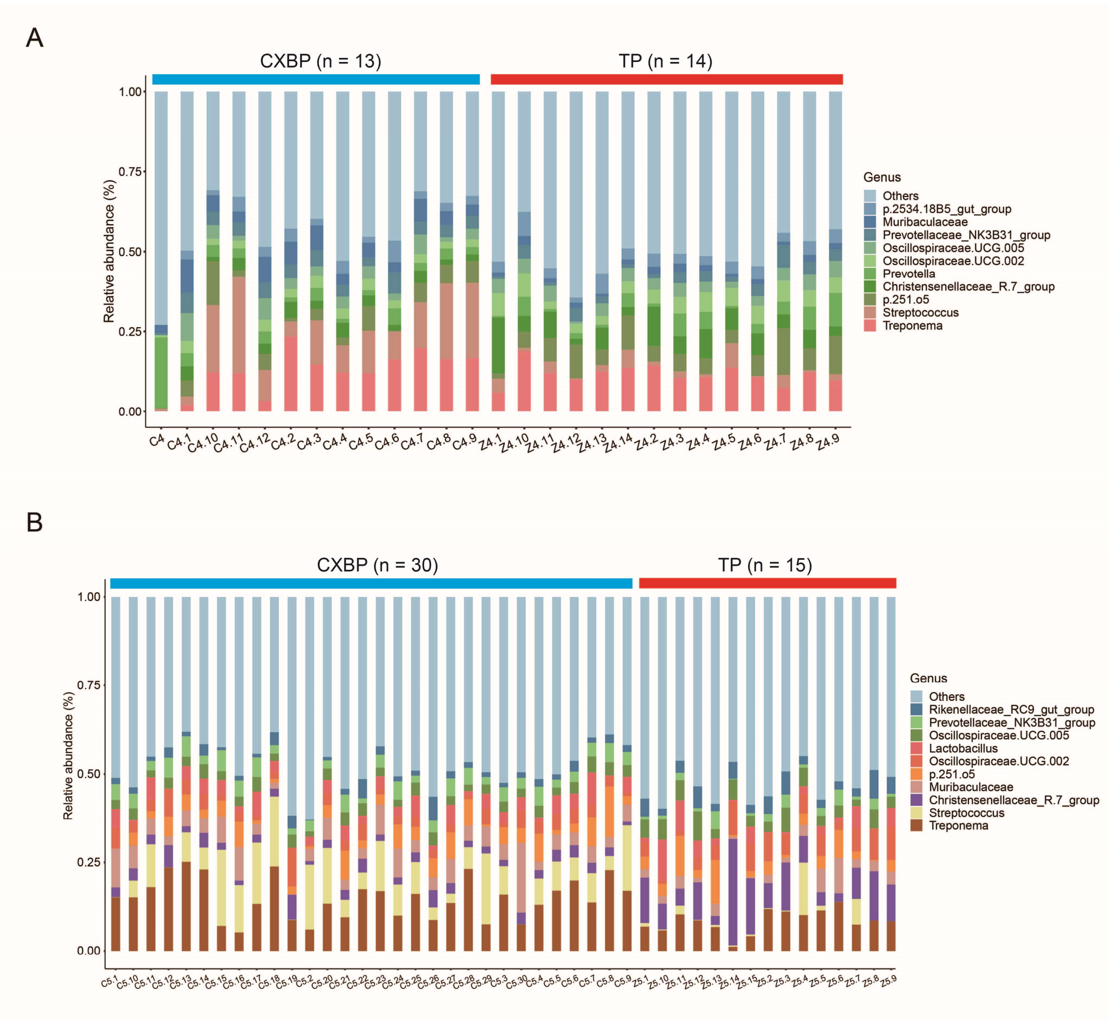

Supplementary Figure 2. The microbial composition of the fecal microbiota at the genus level. (A) The top 10 microbes in terms of relative abundance at the genus level in the feces of pigs at the young adult stage (10 months); genera with a lower relative abundance are classified as “Others”. (B) The top 10 microbes in terms of relative abundance at the genus level in the feces of pigs at the adult stages (2 years); genera with a lower relative abundance are classified as “Others”. CXBP, Chuanxiang black pig. TP, Tibetan pig. C4 and C4.1-C4.12, 10 months old Chuanxiang black pig. Z4.1-Z4.14, 10 months old Tibetan pig. C5.1-C5.30, 2 years old Chuanxiang black pig. Z5.1-Z5.15, 2 years old Tibetan pig.

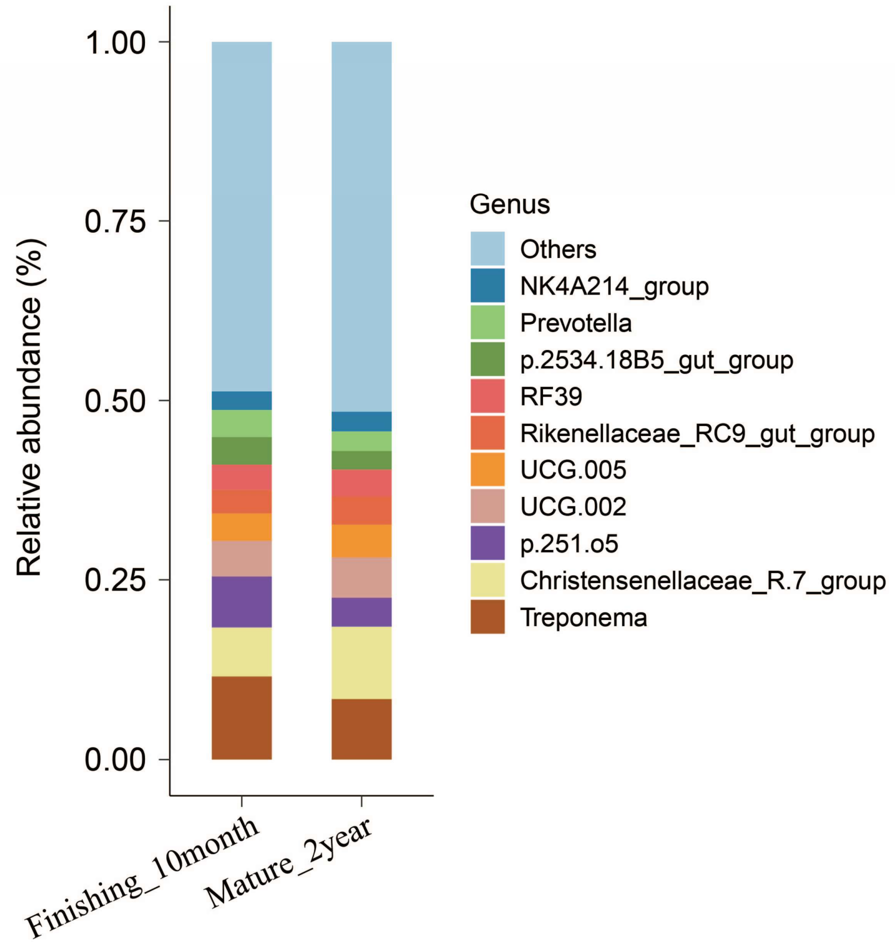

Supplementary Figure 3. The top 10 most abundant microbes in fecal samples of Tibetan pigs at 10 months and 2 years of age at the genus level. Genera with lower relative abundance are classified as “Others”.

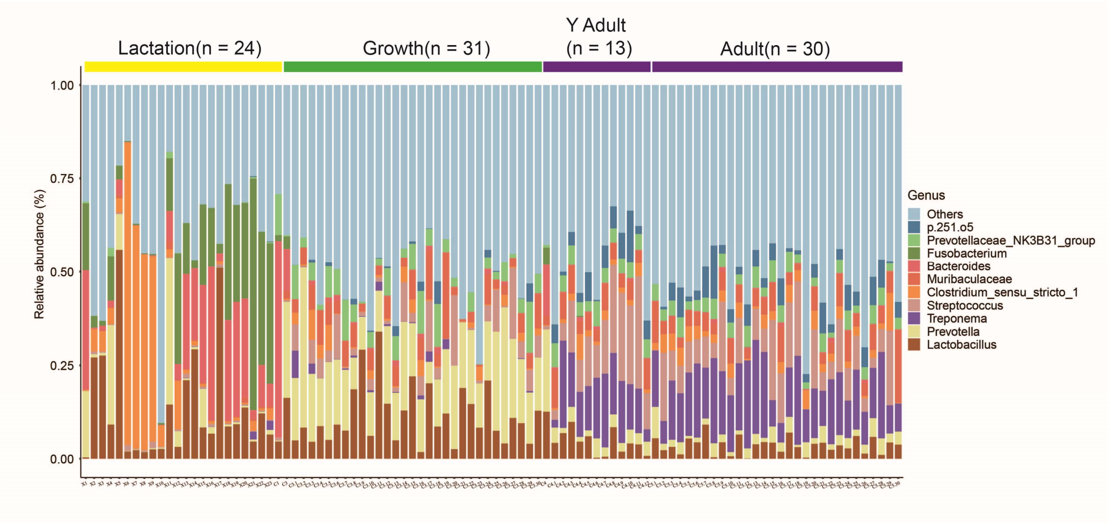

Supplementary Figure 4. The top 10 microbes in terms of relative abundance in the feces of Chuanxiang black pigs at different developmental stages; genera with a lower relative abundance are classified as “Others”. C1 and X1-X23, Chuanxiang black pig at the lactation stage. C3 and C3.1-C3.30, Chuanxiang black pig at the growth stage. C4 and C4.1-C4.12, Chuanxiang black pig at the young adult stage. C5.1-C5.30, Chuanxiang black pig at the adult stage.

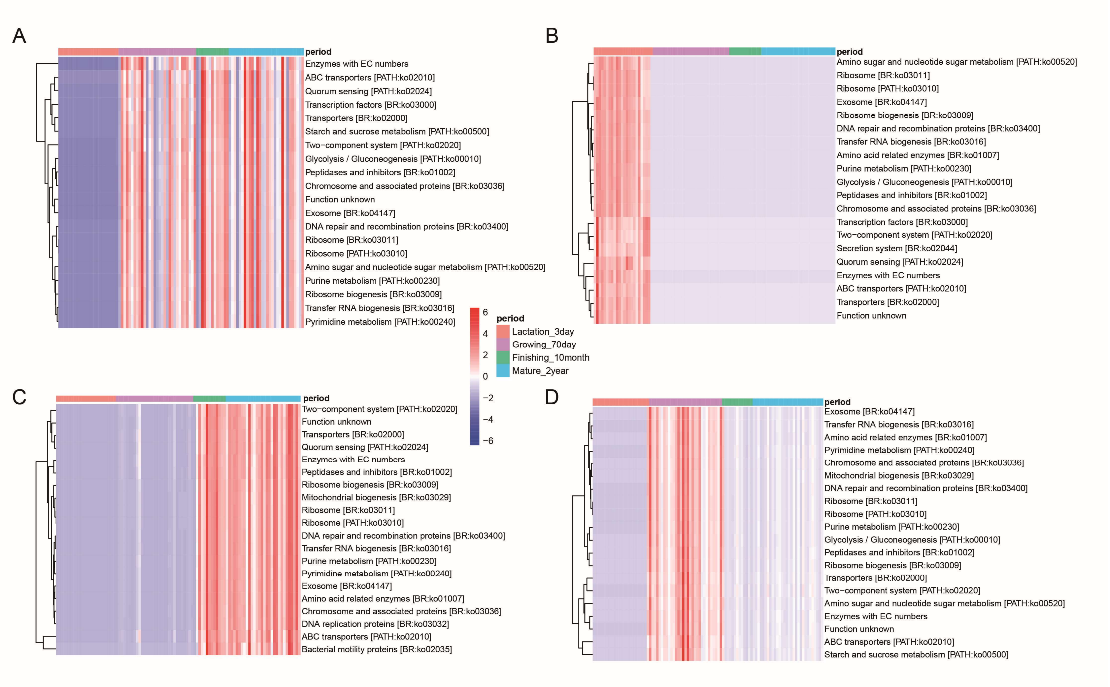

Supplementary Figure 5. After the rich feature was aggregated into four patterns, the top 20 significantly enriched KEGG pathways were found in patterns 1(A), 2(B), 3(C), and 4(D).
